# Down the rabbit hole: how complex do eco-physiological models need to be?

**DOI:** 10.1093/conphys/cox015

**Published:** 2017-02-23

**Authors:** Sean Tomlinson

**Affiliations:** 1 Department of Environment and Agriculture, Curtin University, Bentley, WA 6102, Australia;; 2 Science Directorate, Kings Park and Botanic Gardens, West Perth, WA 6005, Australia

Global temperatures are continuing to rise, and the extreme changes that marine intertidal ecosystems face may make them some of the most susceptible to ocean warming. But how do scientists assess risks to these ecosystems? Many scientists build models. Kish *et al*. (2016) examined an intertidal mussel found along the west coast of North America. They attempted to model body temperatures of these mussels, but found that modelling using average air temperatures alone provided poor estimates of body temperatures. Rather, they needed a complex biophysical model to even come close. While Kish's team found that modelling mussels’ response to temperature was not simple, their most valuable contribution relates to modelling in general.

Kish *et al*. tested their models using a model skills test—a similar approach that meteorologists use when determining how much data are required to predict the weather. Kish's team found that ‘none’ of their models were particularly good at predicting mussel body temperatures at *any* of their field sites. A perfect model would give a reliability score of 1.0, but their models were typically in the area of 0.3. Even more disturbing, their models were least reliable at high temperatures.

Modelling has become the go-to tool for estimating how organisms or ecosystems will respond to changing environments, especially in the face of global climate change. Everybody wants to build models because they can use them to visualize the impacts concisely and intuitively, often with a colourful map or figure. Kish *et al*. found that more complex models were better at predicting mussel body temperatures, but so what? Does increasing data resolution and adding complexity always increase model accuracy? And, at what point does a more complex model cease to give us more accurate estimates—a point of diminishing returns?

How far down this never-ending rabbit hole do we need to go?

In pursuit of a perfect estimate of how an organism or ecosystem will respond to environmental change, models often become so complicated, time consuming and data hungry that they are increasingly misinterpreted as results. Moreover, sometimes scientists can have too much data and go too deep into modelling something that does not necessarily address the complex questions that they initially set out to ask. The model skill tests that Kish *et al*. have explored finally offer our community a way to determine how reliable—or unreliable—our models actually are. Validating these models with independent data is the first step to being able to use these models for practical outcomes. Intertidal mussels provide an interesting study system, but the really important contribution from Kish *et al*. is a timely reminder that, no matter how finely resolved and well informed, models are still hypotheses. In their own words, this is about ‘the ability of a model to predict a series of defined events’, and they provide new tools for our community to test this.

**Figure:**
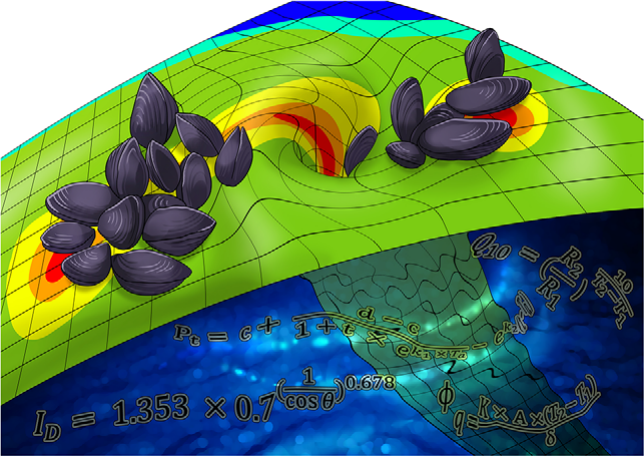

Illustration by Erin Walsh; Email: ewalsh.sci@gmail.com

## References

[cox015C1] Kish NE, Helmuth B, Wethey DS (2016) Physiologically grounded metrics of model skill: a case study estimating heat stress in intertidal populations. Conserv Physiol 4: cow063. 27729979 10.1093/conphys/cow038 PMC5055285

